# Modification of the HIV-1 Fusion Inhibitor 2P23 via N-Glycosylation Sites Enhances Its Cell Membrane Level and Antiviral Activity

**DOI:** 10.3390/ijms27219695

**Published:** 2026-10-29

**Authors:** Sofiia E. Borovikova, Anna V. Tvorogova, Mikhail V. Shepelev, Natalia A. Kruglova

**Affiliations:** Center for Genome Research, Institute of Gene Biology, Russian Academy of Sciences, 34/5 Vavilova Str., 119334 Moscow, Russia; annatvor@mail.ru (A.V.T.); mshepelev@mail.ru (M.V.S.)

**Keywords:** HIV-1 fusion inhibitors, 2P23 peptide, MT-C34 peptide, N-glycosylation, HIV-1 infection, gene therapy

## Abstract

HIV (Human immunodeficiency virus) gene therapy represents a promising strategy to suppress viral replication and potentially achieve long-term control of infection. One of the effective strategies is based on the generation of the genetically modified virus-resistant T cells by expressing peptide inhibitors of viral fusion on the cell membrane. These peptides, such as MT-C34 and 2P23, are derived from HIV gp41 protein and inhibit HIV entry into modified cells. We previously demonstrated in cell-to-cell viral transmission experiments that the 2P23 peptide showed lower protective activity than the MT-C34 peptide. In this study, we hypothesized that the difference in protective activity may arise from the differential N-glycosylation of the peptides. To test this, the 2P23 sequence was modified by introducing the N-glycosylation site (NDT) at the N-, C-, or both termini. These modifications significantly enhanced the plasma membrane expression and protective activity of the 2P23 peptide, with the NDT-2P23-NDT variant being the most potent. The validity of this peptide engineering approach was confirmed with the highly similar HP23L peptide, demonstrating an increased cell membrane expression and antiviral potency of the modified variant. Experiments with the N-glycosylation inhibitor tunicamycin provide indirect evidence that this effect is glycosylation-dependent. These findings suggest that introducing N-glycosylation sites is a versatile approach to improve the potency of membrane-bound antiviral peptides for HIV gene therapy.

## 1. Introduction

Despite the advancements in antiretroviral therapy, HIV infection remains a global public health challenge. One of the promising therapeutic approaches involves the generation of genetically modified virus-resistant cells through the surface expression of peptide fusion inhibitors [1].

Peptide fusion inhibitors are short synthetic peptides derived from the N- or C-terminal heptad repeat domains (NHR and CHR, respectively) of the HIV gp41 protein. These peptides disrupt the conformational changes required for viral-cellular membrane fusion, thereby blocking viral entry and preventing infection progression. Notably, C-peptides exhibit superior antiviral potency owing to their enhanced solubility and are more widely utilized in the development of therapeutic strategies [2]. To date, two soluble peptide fusion inhibitors have been approved for clinical use: enfuvirtide [3] and albuvirtide [4]. However, their utility is limited by the requirement for frequent injections, adverse side effects, and the emergence of drug-resistant viral strains [5]. Next-generation peptides, such as MT-C34 and 2P23, exhibit significantly higher potency and a broader spectrum of activity, including that against enfuvirtide-resistant HIV isolates [6]. The MT-C34 sequence is directly derived from the CHR domain of gp41 and was developed by including two critical upstream residues, methionine and threonine, to the N-terminus of the C34, an earlier variant of peptide fusion inhibitors [7]. This modification drastically enhanced the peptide’s inhibitory potency by establishing the M-T hook structure, which is vital for overcoming drug resistance. In contrast to MT-C34, the 2P23 peptide incorporates extensive structural modifications relative to the original gp41 sequence, including not only the M-T hook motif, but also an N-terminal capping residue, amino acid substitutions optimizing intrahelical salt bridges, and critical residues required for HIV-2 binding [8]. These modifications cooperatively enhanced the peptide’s overall target-binding affinity across divergent HIV variants, ultimately driving its highly potent fusion-inhibitory activity against T-20-resistant HIV-1 strains, HIV-2, and SIV [8]. Currently, the 2P23 peptide is under active investigation as a therapeutic agent when conjugated to an anti-CCR5 antibody [9], and it has also been evaluated as a prophylactic agent [10]. Furthermore, we adapted 2P23 as an HIV gene therapy modality, demonstrating high efficacy upon membrane-bound expression in target cells [11].

HIV gene therapy aims to achieve a functional cure—a condition when viral levels remain below detection limits without the need for combination antiretroviral therapy (cART)—by replacing the latent HIV reservoir with virus-resistant, genetically modified cells. To date, prominent gene therapy strategies for generating HIV resistance include host coreceptor knockout [12,13] and the expression of protective antiviral factors, such as cellular restriction factors [14,15] and peptide fusion inhibitors [11,16], introduced through viral transduction or targeted knock-in mechanisms [17]. Autologous CD4^+^ T cells and especially hematopoietic stem/progenitor cells (HSPCs) serve as the primary cellular targets for such interventions, with the predominant strategy relying on their ex vivo modification followed by subsequent infusion into the patient [18,19]. For HSPCs, conditioning is typically required prior to infusion to clear bone marrow niches [20]. While targeting CD4^+^ T cells is technically simpler, their limited lifespan necessitates repeated infusions. Conversely, HSPCs self-renew to provide a sustained supply of HIV-resistant cells, offering a potential single-dose functional cure.

Within this landscape, genetically encoded, membrane-anchored gp41-derived peptides represent an especially promising strategy for HIV gene therapy [17]. The C34 and C46 peptides have been evaluated in preclinical [21] and clinical settings [22,23] and have demonstrated a favorable safety profile (ClinicalTrials.gov: NCT03020524, NCT01734850, and NCT03593187). Concurrently, these studies revealed that a low baseline percentage of modified cells, sub-optimal engraftment, and poor long-term persistence remain key barriers to therapeutic success [22,23]. This low percentage of gene-modified cells in vivo likely explains the failure of such approaches to maintain viral HIV RNA levels below the detection limit following the interruption of cART.

No preclinical or clinical evaluation data are currently available for the 2P23 peptide. Importantly, like other HIV-1 gp41-derived peptides, 2P23 was initially developed and optimized in a soluble form, and subsequently modified with lipid moieties [24,25] or conjugated to monoclonal antibodies [9,26] for membrane targeting. While these modified analogs offer improved local retention at the cell surface, only genetically encoded membrane bound peptides could be employed in gene therapy. Therefore, systematic studies are strictly required to maximize 2P23 plasma membrane expression and therapeutic potency within a membrane-bound context.

Previously, we developed and optimized genetic constructs for the membrane expression of peptide fusion inhibitors, including MT-C34 and 2P23, in human cells [11]. In cell-to-cell infection assays using Raji and Raji/CD4/CCR5 cell cultures expressing the MT-C34 peptide, complete protection of target cells was observed against HIV pseudoviruses. However, protection mediated by 2P23 was not as effective as that of MT-C34 [11]. We hypothesized that the distinct protective efficacies of these peptides might arise from sequence differences leading to variations in cell membrane expression levels and/or stability on the cell membrane. According to predictions by the NetNGlyc web tool [27], the MT-C34 peptide contains a potential N-glycosylation site that is absent in 2P23. Previous studies suggest that N-glycosylation protects proteins from proteolytic degradation [28] and inhibits cell-surface internalization [29], thereby increasing protein half-life on the plasma membrane. Modifications of N-glycosylation sites are known to modulate protein stability, where the removal of these sites reduces stability, while their restoration or introduction significantly enhances functional activity [30,31].

In this study, we introduced N-glycosylation sites (NDT) at the N-terminus, C-terminus, or both termini of the 2P23 peptide, generating NDT-2P23, 2P23-NDT, and NDT-2P23-NDT. To evaluate the protective properties of these novel peptides, we generated T cell lines expressing the NDT-tagged peptides and performed cell-to-cell viral infection assays using pseudoviruses with CCR5- and CXCR4-tropic HIV envelopes. Our results demonstrate that N-glycosylation significantly increases the membrane surface expression and antiviral potency of 2P23 variants. Furthermore, we successfully extended this engineering strategy to the structurally similar fusion inhibitor peptide HP23L, which yielded similar improvements in both surface level and protective efficacy. Overall, this study establishes the introduction of N-glycosylation sites as a versatile and effective approach for optimizing membrane-bound antiviral peptides, offering a promising strategy for enhancing the efficiency of HIV gene therapy.

## 2. Results

### 2.1. Modification of the 2P23 Peptide with N-Glycosylation Sites

**Figure 1:**
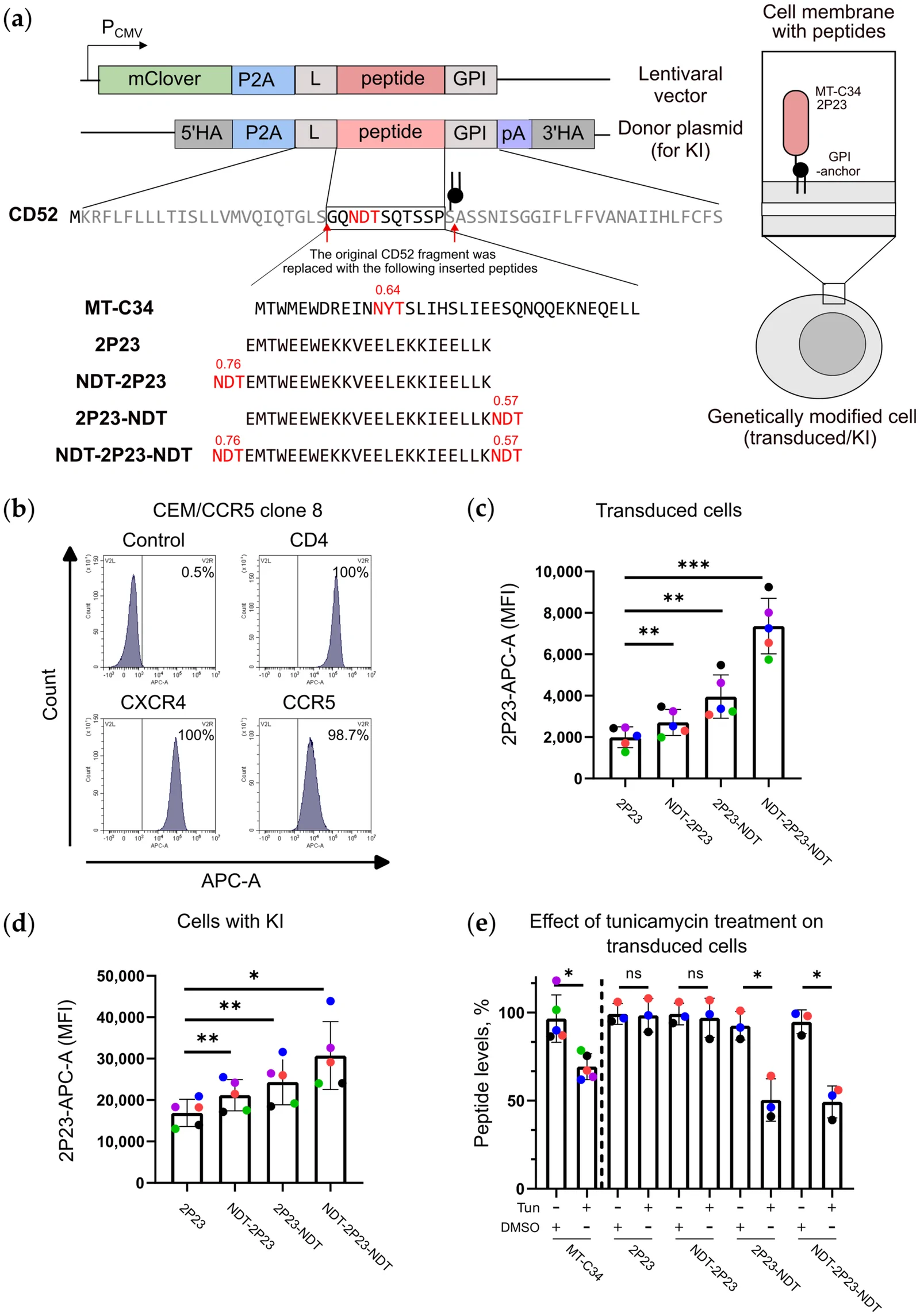
Characterization of peptides containing potential N-glycosylation sites. (**a**) Schematic representation of the pUCHR-mClover lentiviral vector and the donor vector for knock-in into the *CXCR4* gene. P_CMV_, cytomegalovirus promoter; P2A, ribosome skipping signal; L and GPI, leader sequence and GPI-anchor attachment signals derived from CD52; 5′HA and 3′HA, homology arms for exon 2 of the *CXCR4* gene; pA, polyadenylation signal; KI—knock-in. Proteolytic cleavage sites are indicated by arrows; the serine residue upstream of the second cleavage site is modified by the GPI-anchor. Predicted N-glycosylation sites (NetNGlyc 1.0) are highlighted in red, with model-derived glycosylation scores indicated above. In the NetNGlyc software, the potential glycosylation value represents the mean score predicted by an ensemble of nine independent artificial neural networks. The scheme on the right shows the peptide on the cell membrane. (**b**) Flow cytometry analysis of HIV entry receptor and coreceptors on CEM/CCR5 clone 8 cells. (**c**,**d**) Flow cytometry analysis of cell-surface 2P23 and NDT-peptides on CEM/CCR5 clone 8 cells (**c**) transduced with lentiviruses and sorted by mClover expression, or (**d**) harboring the peptide knock-in into exon 2 of *CXCR4* and sorted for peptide expression. MFI, median fluorescence intensity in the APC channel of the entire sorted population. Data points from individual experiments are color-coded. For panels (**c**,**d**) statistical significance between multiple groups was evaluated using repeated-measures analysis of variance (RM-ANOVA). To account for potential violations of the sphericity assumption, the Greenhouse–Geisser correction was applied, followed by Dunnett’s post hoc test for multiple comparisons against the 2P23 control. (**e**) Evaluation of NDT-peptides glycosylation using the N-glycosylation inhibitor tunicamycin. Transduced CEM/CCR5 clone 8 cells expressing the peptides were cultured for 5 days in medium supplemented with either tunicamycin (Tun) or DMSO, followed by surface staining for the peptide. The dashed line separates MT-C34 experiments performed separately. The median APC signal of DMSO-treated or tunicamycin-treated cells was normalized to the signal of stained control cells cultured in unmodified medium. Pairwise statistical comparisons between two related groups (tunicamycin-treated versus DMSO-treated samples) were performed using a ratio paired *t*-test. *p*-values are categorized by significance thresholds as follows: (*) *p* < 0.05, (**) *p* < 0.01, (***) *p* < 0.001, (ns)—not significant.

We previously showed that membrane-anchored HIV inhibitory peptide MT-C34 was more effective than 2P23 at preventing cell-to-cell HIV transmission in a Raji cell model [11]. We reasoned that the difference in expression levels and protective properties between MT-C34 and 2P23 might arise from the difference in N-glycosylation. Therefore, to enhance the cell-surface stability and protective efficacy of the membrane-anchored peptide 2P23, the MT-C34 and 2P23 peptide sequences were searched for canonical N-glycosylation sites using the NetNGlyc-1.0 web tool [27]. This analysis revealed that the MT-C34 peptide contains a potential N-glycosylation motif (NYT), whereas no such sites were identified within the 2P23 sequence (Figure 1a). Based on this finding, we hypothesized that introducing additional N-glycosylation sites into the 2P23 sequence could enhance the peptide stability on the cell surface, thereby increasing its membrane accumulation. To preserve the core peptide sequence and maintain its protective efficacy, modifications were restricted to the N- and C-terminal regions [32].

To direct peptides to the plasma membrane, our previously developed expression constructs were utilized wherein the peptide sequence is embedded within the structural context of the CD52 molecule [32] (Figure 1a). In this system, translation yields an N-terminal signal peptide that directs the nascent chain to the endoplasmic reticulum, followed by the target peptide sequence, and a C-terminal glycosylphosphatidylinositol (GPI) anchoring signal that tethers the peptide to the plasma membrane. Following post-translational processing, peptides are displayed on the plasma membrane via a GPI anchor attached to the C-terminal serine residue devoid of any additional CD52-derived amino acid residues. By utilizing this framework, the engineered modification sites were positioned at the junctions between the 2P23 peptide and the flanking CD52 sequences (Figure 1a).

The canonical consensus sequence for N-linked glycosylation is defined as N-X-S/T, where X represents any amino acid except proline [33]. To select a specific motif, we analyzed the primary structure of native CD52, which contains a functional GQNDT sequence at the site where the peptide variants are inserted (Figure 1a). The data indicate that this substituted NDT motif harbors a functional N-glycosylation site [32,34]. Based on these findings, the NDT tripeptide was selected as the candidate N-glycosylation motif.

### 2.2. Introduction of N-Glycosylation Sites into the 2P23 Peptide Enhances Its Cell-Surface Expression in the CEM T-Cell Line

To evaluate the impact of the introduced modifications on the cell-surface expression and protective efficacy of 2P23, we generated stable cell lines using lentiviral transduction or CRISPR/Cas9-mediated knock-in into the *CXCR4* gene locus [11]. These lines were engineered to express variants NDT-2P23, 2P23-NDT, and NDT-2P23-NDT and control peptides MT-C34 and 2P23 on the basis of previously described CCRF-CEM T-cell line stably overexpressing the CCR5 coreceptor (hereafter referred to as CEM/CCR5 clone 8) [35]. This particular cell line was chosen due to its high endogenous expression of CD4 and CXCR4, which are essential for downstream HIV infection assays (Figure 1b). Lentiviral vectors were constructed on the basis of the pUCHR-mClover backbone [11], where the peptide sequences were linked to an mClover reporter via a P2A self-cleaving peptide thus allowing to monitor transduction efficiency and cell sorting (Figure 1a). Transduction was performed at a multiplicity of infection (MOI) of less than 0.3, which ensured single-copy proviral integration and prevented artificial upregulation of peptide expression due to multiple proviral integration events. Stably transduced cell pools (accounting for approximately 30% of mClover-positive events) were subsequently enriched via fluorescence-activated cell sorting (FACS) (Supplementary Figure S1a).

To quantify the resulting peptide density on the plasma membrane, the sorted lentiviral lines were stained with a polyclonal anti-2P23 antibody and analyzed by flow cytometry (Figure 1c). All cell lines expressing NDT-modified 2P23 variants exhibited substantially higher surface expression of peptides as compared to the parental non-modified 2P23 variant. Specifically, the median fluorescence intensity (MFI) of the sorted populations was as follows (mean ± standard deviation (SD)): 7363 ± 1345 for NDT-2P23-NDT, 3957 ± 1048 for 2P23-NDT, 2709 ± 629 for NDT-2P23, and 1988 ± 504 for 2P23 (Figure 1c). Notably, the insertion of two glycosylation sites in the NDT-2P23-NDT variant led to more than a 3-fold increase in the membrane expression relative to the unmodified 2P23 peptide.

The second approach to generate cells stably expressing 2P23 variants relied on CRISPR/Cas9-mediated knock-in into exon 2 of the *CXCR4* gene, allowing us to evaluate peptide expression under the control of the endogenous promoter (Figure 1a). In this system, successful peptide knock-in is accompanied by *CXCR4* knockout. The cassette design directing peptide export to the cell surface was identical to that used in the lentiviral vectors. CEM/CCR5 clone 8 cells were co-electroporated with three plasmids encoding the donor template, Cas9 nuclease, and sgRNA, respectively. Flow cytometry confirmed that the efficiencies of peptide knock-in and concomitant *CXCR4* knockout were highly consistent across all experimental groups (Supplementary Figure S1b–d). Following FACS enrichment based on the peptide surface signal (Supplementary Figure S1e), the highest membrane density was again detected for the NDT-2P23-NDT variant, yielding an average population MFI of 30,744 ± 8189. The single-site glycosylation variants also displayed enhanced surface accumulation as compared to the unmodified control: 2P23-NDT (MFI = 24,340 ± 5492), NDT-2P23 (MFI = 21,173 ± 3784), and 2P23 (MFI = 16,890 ± 3284) (Figure 1d). Taken together, these results demonstrate that the introduction of N-glycosylation sites substantially increased peptide levels on the plasma membrane, with the NDT-2P23-NDT variant consistently achieving the highest surface density in both experimental models.

### 2.3. The N-Glycosylation Inhibitor Tunicamycin Reduces the Cell-Surface Levels of MT-C34, 2P23-NDT, and NDT-2P23-NDT Peptides

To confirm the contribution of N-glycosylation to the enhanced cell membrane exposure of the NDT-modified variants, the N-glycosylation inhibitor tunicamycin was employed. Transduced CEM/CCR5 cells expressing the respective peptides were incubated in the medium supplemented with either tunicamycin or DMSO (vehicle control) for 5 days, followed by flow cytometry analysis to quantify cell-surface peptide levels. In the experiments described above, MT-C34 peptide staining in both transduction and knock-in settings was performed using anti-MT-C34 monoclonal antibodies (Supplementary Figure S2a). However, in experiments involving tunicamycin, polyclonal anti-MT-C34 antibodies were employed (Supplementary Figure S2b), given that the difference in post-translational modifications may affect antibody-peptide binding. Similarly, the 2P23 peptide was detected using polyclonal antibodies.

Cells expressing MT-C34, 2P23-NDT, and NDT-2P23-NDT exhibited a statistically significant decrease in the peptide MFI levels following tunicamycin treatment. In contrast, no changes were observed for the parental 2P23 and the single-modified NDT-2P23 variant (Figure 1e). These data suggest that the C-terminal NDT site is likely the primary site for N-glycosylation in the modified 2P23 variants.

Next, we evaluated the effect of tunicamycin treatment on the 2P23 peptide levels in cells with knock-in into the *CXCR4* locus. Unexpectedly, cell surface levels of 2P23 and its modified versions increased upon tunicamycin treatment (Supplementary Figure S2c). These data suggest that *CXCR4* gene expression or CXCR4 protein cell membrane levels depend on N-glycosylation. Flow cytometry confirmed that tunicamycin treatment elevated cell-surface CXCR4 protein levels (Supplementary Figure S2d). We conclude that the process of CXCR4 protein maturation and membrane expression is negatively regulated by N-glycosylation. Therefore, in a CXCR4 knock-in model we were unable to confirm the role of N-glycosylation in promoting higher membrane levels of modified 2P23 peptide variants. Consequently, these data were excluded from further analysis due to the confounding effects of endogenous regulatory processes.

Nevertheless, the data from experiments using transduced cells clearly indicate that inhibition of N-glycosylation significantly impairs the membrane accumulation of the MT-C34, 2P23-NDT, and NDT-2P23-NDT variants, thereby providing indirect evidence of a functional link between N-glycosylation and HIV fusion inhibitory peptide cell-surface density.

### 2.4. NDT-Modified Peptides Provide Comparable or Superior Protection to Target Cells in Cell-to-Cell Virus Transmission Assays Relative to 2P23

**Figure 2:**
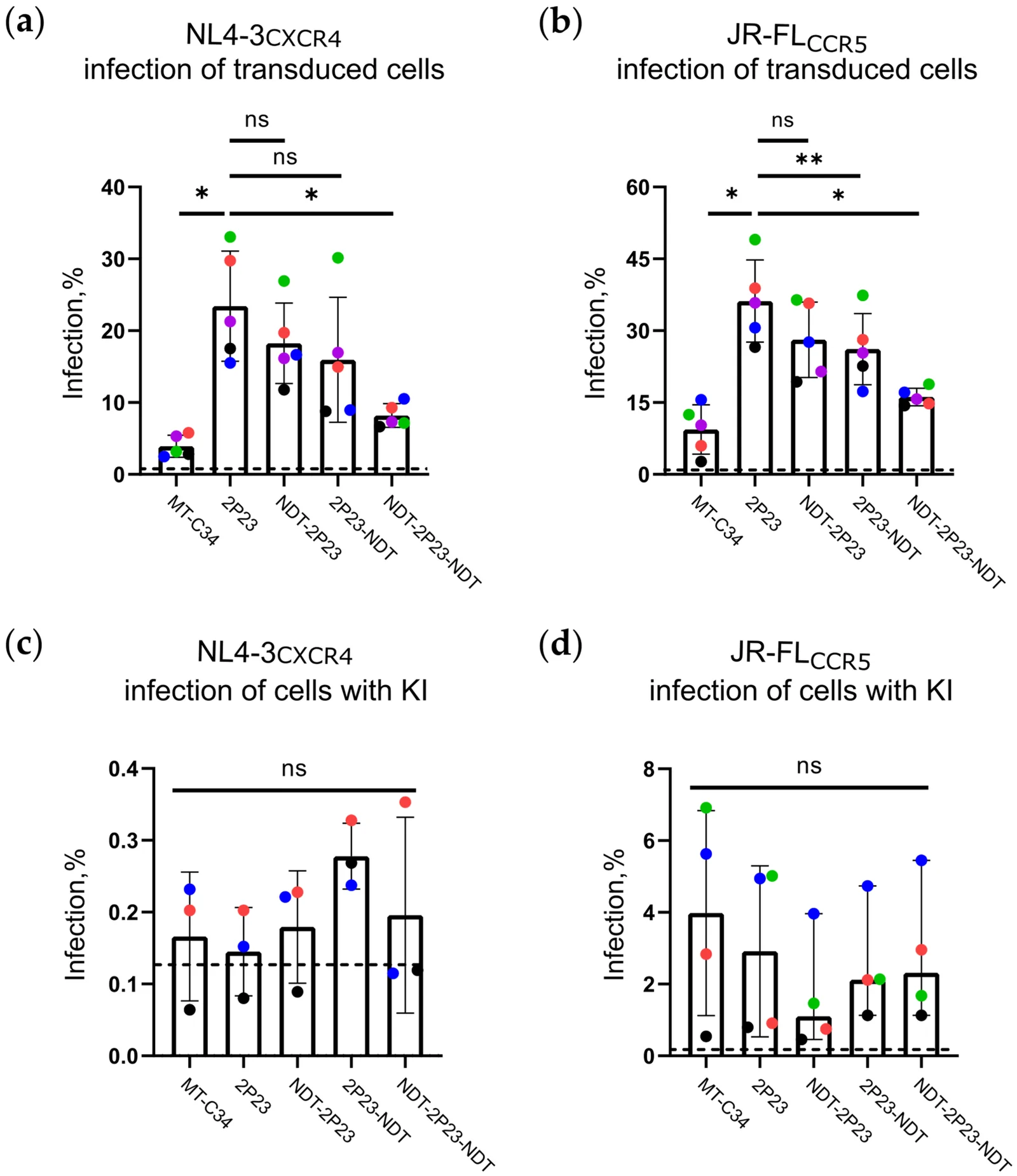
Protective properties of NDT peptides in cell co-culture infection assay. (**a**,**b**) Cell co-culture assays evaluating cell-to-cell transmission of pseudoviruses harboring (**a**) the CXCR4-tropic NL4-3 envelope or (**b**) the CCR5-tropic JR-FL envelope in transduced CEM/CCR5 clone 8 cells expressing different peptides. (**c**,**d**) Co-culture infection assays utilizing (**c**) NL4-3 or (**d**) JR-FL envelopes in CEM/CCR5 clone 8 cells with knock-in. Raji cells were electroporated with plasmids for pseudovirus assembly and co-cultured with target cells at a 1:1 ratio; 72 h after Raji electroporation, infection levels were assessed by measuring the luciferase reporter signal. Luciferase activity in peptide-expressing cells was normalized to that of control cells lacking the peptide. The dashed line indicates the background luciferase activity, determined by co-culturing pseudovirus-producing Raji cells with parental Raji cells. Data points from individual experiments are color-coded. Statistical significance between multiple groups was evaluated using RM-ANOVA. To account for potential violations of the sphericity assumption, the Greenhouse–Geisser correction was applied, followed by Dunnett’s post hoc test for multiple comparisons against the 2P23 control. *p*-values are categorized by significance thresholds as follows: (*) *p* < 0.05, (**) *p* < 0.01, (ns)—not significant.

To evaluate the protective efficacy of the NDT-peptides, we performed cell-to-cell virus transmission assays utilizing an inverted luciferase reporter system [11,36] and HIV-1 envelopes from either the CXCR4-tropic NL4-3 strain (Figure 2a) or the CCR5-tropic JR-FL strain (Figure 2b). Raji producer cells (which express very low levels of CD4 and CCR5; Supplementary Figure S3a) were co-cultured with lentivirus-transduced CEM/CCR5 clone 8 target cells, and the efficiency of infection was quantified via the luciferase signal. In the producer cells, the luciferase gene is positioned in an inverted orientation and interrupted by an intron, rendering it inactive. Upon pseudovirus assembly, the intron is spliced out. Following entry into target cells, the restored luciferase gene is actively expressed. The background luciferase activity was determined by co-culturing pseudovirus-producing Raji cells with parental Raji cells. MT-C34-transduced cells exhibited the lowest infection levels (averaging 4% for NL4-3 and 9.5% for JR-FL), in contrast to the 2P23-expressing cells, which showed high infection rates (averaging approximately 23% for NL4-3 and 36% for JR-FL). For the four 2P23-based peptide variants, a negative correlation between mean infection rates and cell-surface MFI was observed (Pearson’s r = −0.97 for NL4-3 and r = −0.95 for JR-FL; *p* < 0.05), indicating that higher surface peptide levels strictly correlate with decreased viral transmission (Supplementary Figure S3b,c). Specifically, the NDT-2P23-NDT variant exhibited the highest membrane density and demonstrated the most potent inhibition, reducing NL4-3 and JR-FL infection levels to 8% and 16%, respectively (Figure 2a,b).

In cell-to-cell infection assays using cells with knock-in, the infection rates did not exceed 0.35% for NL4-3 and 7% for JR-FL HIV strains (Figure 2c,d). No statistically significant differences were observed between the various knock-in peptide samples, regardless of the viral envelope used. Upon infection with the NL4-3 strain, the infection rate remained at background levels (<1%) across all knock-in cell types (Figure 2c). This background-level infection, which was independent of the surface-displayed peptide variant, is mediated by the simultaneous knockout of the endogenous *CXCR4* gene, whose product serves as the essential coreceptor for NL4-3 CXCR4-tropic HIV entry.

Because the engineered cell lines were generated using multiple sequential sorting steps, we sought to confirm that they maintained comparable pseudoviral infection efficiencies. To confirm that, control infection assays were performed using the vesicular stomatitis virus G (VSV-G) envelope. VSV-G utilizes the low-density lipoprotein receptor (LDL-R), which is ubiquitously expressed in CEM/CCR5 cells and is not targeted by gp41-derived peptides. No significant differences in infection efficiency were observed between the cell lines, regardless of the presence of antiviral peptides on the cell surface (Supplementary Figure S3d,e).

Taken together, these infection assays demonstrate a clear correlation between peptide surface density and protective efficacy in the transduced cell model, where the NDT-2P23-NDT variant consistently achieved the most potent viral inhibition (Supplementary Figure S3b,c).

### 2.5. Evaluating Alternative N-Glycosylation Motifs and Structural Generalizability Using the HP23L Peptide

**Figure 3:**
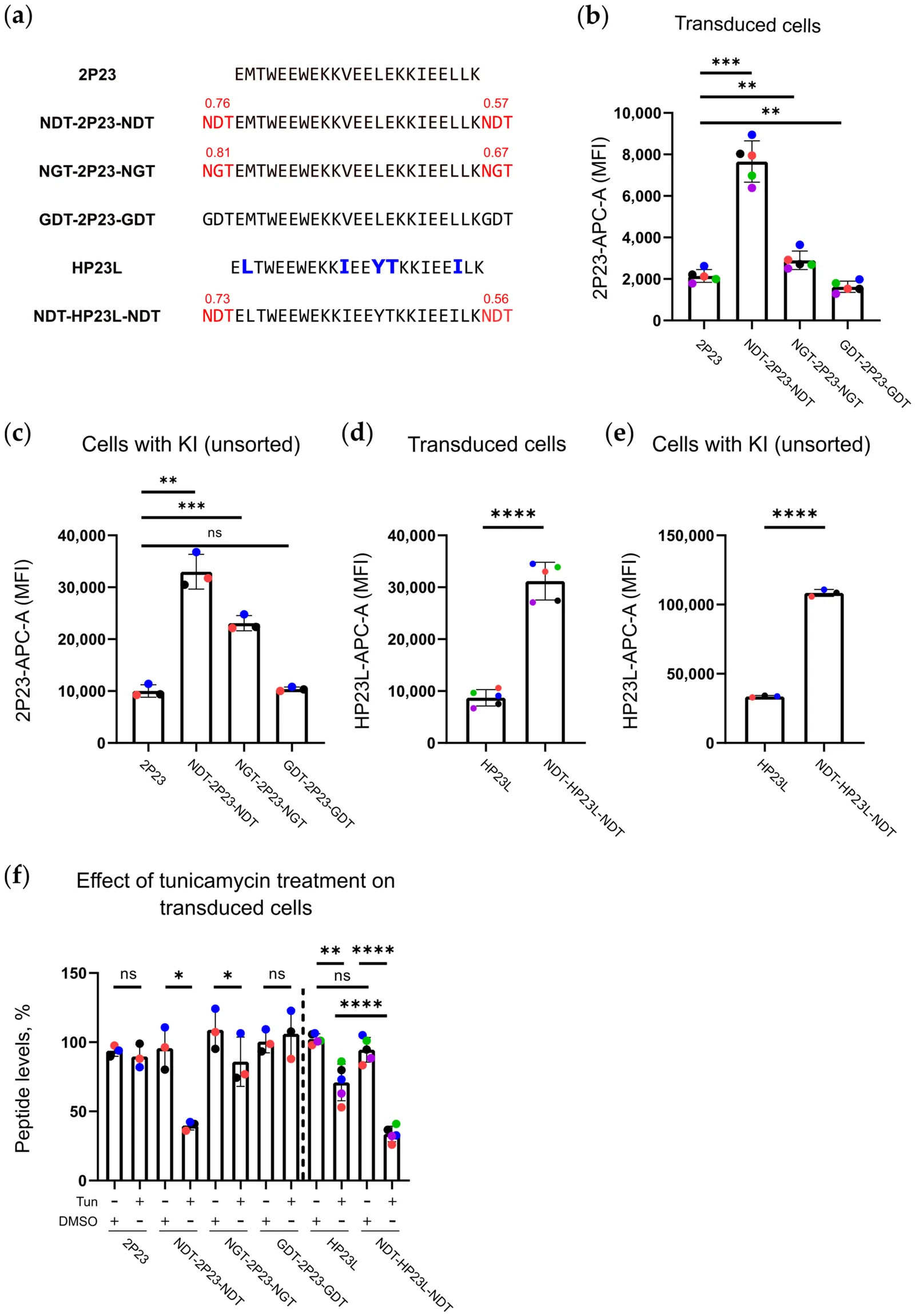
Evaluation of alternative peptide variants: NGT-2P23-NGT, GDT-2P23-GDT, HP23L, and NDT-HP23L-NDT. (**a**) Amino acid sequences of the engineered peptides. Predicted N-glycosylation sites (NetNGlyc-1.0) are highlighted in red, with corresponding model-derived glycosylation scores indicated above. The 2P23 and HP23L sequences are highly similar. Amino acid residues in HP23L differing from 2P23 are indicated in bold blue. (**b**–**e**) Flow cytometry analysis of cell-surface peptide expression of 2P23-variants (**b**,**c**) and HP23L-variants (**d**,**e**). For panels (**b**,**d**), CEM/CCR5 clone 8 cells were transduced with lentiviruses and sorted based on mClover expression. For panels (**c**,**e**), CEM/CCR5 clone 8 cells were analyzed following CRISPR/Cas9 knock-in into exon 2 of the *CXCR4* gene. Five days after electroporation, peptide MFI was evaluated by gating exclusively on the knock-in population. Data points from individual experiments are color-coded. For 2P23-variants (**b**,**c**) statistical significance between multiple groups was evaluated using RM-ANOVA. To account for potential violations of the sphericity assumption, the Greenhouse–Geisser correction was applied, followed by Dunnett’s post hoc test for multiple comparisons against the 2P23 control. For HP23L (**d**,**e**) statistical significance was evaluated using a ratio paired *t*-test. (**f**) Evaluation of alternative peptide variants glycosylation using the N-glycosylation inhibitor tunicamycin. Transduced CEM/CCR5 clone 8 cells expressing the peptides were cultured for 5 days in medium supplemented with either tunicamycin (Tun) or DMSO, followed by surface staining for the peptide. The dashed line separates HP23L and NDT-HP23L-NDT experiments conducted at separate time points. The median APC signal was normalized to the signal in stained control cells cultured in unmodified medium. Pairwise statistical comparisons between two related groups (tunicamycin-treated versus DMSO-treated samples) were performed using a ratio paired *t*-test. For the HP23L variants, a ratio paired *t*-test was also used to compare the DMSO-HP23L versus DMSO-NDT-HP23L-NDT, and Tun-HP23L versus Tun-NDT-HP23L-NDT pairs. *p*-values are categorized by significance thresholds as follows: (*) *p* < 0.05, (**) *p* < 0.01, (***) *p* < 0.001, (****) *p* < 0.0001, (ns)—not significant.

Based on the primary structure of native CD52, we initially selected aspartic acid (D) as the variable residue X within the canonical N-glycosylation consensus sequence (N-X-S/T). However, a comprehensive large-scale screening of glycosylation patterns previously demonstrated that small, uncharged residues—such as glycine (G), valine (V), or serine (S)—are statistically preferred at the X position [33]. According to predictions generated by the NetNGlyc-1.0 web server [27], the probability of N-linked glycosylation for these alternative residues is higher than that for the NDT combination. Specifically, the predicted glycosylation potentials for the N-terminal sites, ranked in descending order, are as follows: 0.8332 for NVT, 0.8147 for NGT, 0.7739 for NST, and 0.7653 for the native NDT motif. The corresponding predicted values for the potential N-glycosylation sites at the C-terminus of 2P23 were 0.7084, 0.6716, 0.6010, and 0.5750, respectively. To compare our NDT-2P23-NDT construct with these theoretically optimized variants, we generated the NGT-2P23-NGT expression cassette (Figure 3a).

Furthermore, to systematically evaluate the specific contribution of N-glycosylation to the phenotypes observed in the modified NDT-2P23-NDT peptide, a control variant named GDT-2P23-GDT was designed, in which the critical asparagine residue was substituted with a glycine residue (Figure 3a). We hypothesized that the cell-surface expression levels, antiviral efficacy, and tunicamycin sensitivity of this non-glycosylatable control should remain comparable to those of the parental 2P23 peptide. Such data would provide definitive evidence confirming the direct role of N-glycosylation in driving the membrane accumulation of NDT-2P23-NDT.

In addition to the analysis of the computationally superior NGT-2P23-NGT variant and the non-glycosylatable GDT-2P23-GDT control, we sought to determine whether the introduction of NDT sites could improve the protective properties of the structurally related peptide HP23L (Figure 3a). We previously demonstrated that HP23L, similar to 2P23, exhibited lower protective capacity compared to MT-C34 [11]. Therefore, to assess the generalizability and scalability of this glycoengineering approach to other fusion inhibitor peptides, expression constructs for both the parental HP23L and the modified NDT-HP23L-NDT variants were generated.

To assess the membrane expression level and peptide protective properties, we generated both lentiviral and knock-in cell lines for the novel peptide variants (NGT-2P23-NGT, GDT-2P23-GDT, HP23L, and NDT-HP23L-NDT) as described above (Figure 3b–e; Supplementary Figure S4a–e).

In the lentiviral transduction model, the NDT-2P23-NDT variant was expressed at the highest level while the NGT-2P23-NGT variant showed a slightly higher surface expression compared to the parental 2P23 peptide (MFI of 2897 ± 445 vs. 2147 ± 308, respectively; Figure 3b). The level of the non-glycosylatable GDT-2P23-GDT control was lower than that of 2P23, averaging 1623 ± 271 MFI (Figure 3b). In cells with knock-in, the surface level of NGT-2P23-NGT was higher than that of 2P23 (Figure 3c) but remained lower than that of NDT-2P23-NDT. The level of non-glycosylatable GDT-2P23-GDT control was comparable to that of the unmodified 2P23 (Figure 3c). Taken together, in contrast to computational prediction, these data indicated that the NDT motif provides higher peptide expression, while NGT is less effective. The addition of the non-glycosylatable GDT site did not increase the peptide levels, confirming that N-glycosylation is required to enhance peptide expression levels at the cell membrane.

Analysis of HP23L peptide modifications showed that similarly to 2P23, introducing the NDT motif into the HP23L backbone significantly enhanced its cell-surface density, driving an approximate 3.5- or 3.3-fold increase over the unmodified HP23L control in transduced cells or in cells with knock-in, respectively (Figure 3d,e).

### 2.6. Tunicamycin Reduces Cell-Surface Levels of NGT- and NDT-Peptides but Does Not Affect the GDT-Variant

To further confirm the role of N-glycosylation in promoting elevated membrane expression, we evaluated the effect of tunicamycin treatment on the surface levels of the novel peptide variants in the transduced cell lines (Figure 3f). For the NGT-2P23-NGT variant, a statistically significant decrease in cell-surface expression was observed upon tunicamycin treatment compared to the DMSO control. However, the magnitude of this reduction was markedly less pronounced than that observed for the NDT-2P23-NDT. Concurrently, the expression levels of the non-glycosylatable GDT-2P23-GDT variant remained unaltered in the presence of tunicamycin. This lack of response indirectly confirms our hypothesis that N-linked glycosylation is required for driving the enhanced membrane accumulation of the NDT-2P23-NDT peptide.

Both HP23L and NDT-HP23L-NDT variants exhibited a decrease in signal intensity in the presence of tunicamycin (Figure 3f). Notably, the magnitude of this reduction was not equivalent, with the double-NDT variant demonstrating a more pronounced decline than the unmodified peptide.

### 2.7. Evaluation of the Protective Efficacy of the NGT- and GDT-Modified Peptide Variants

**Figure 4:**
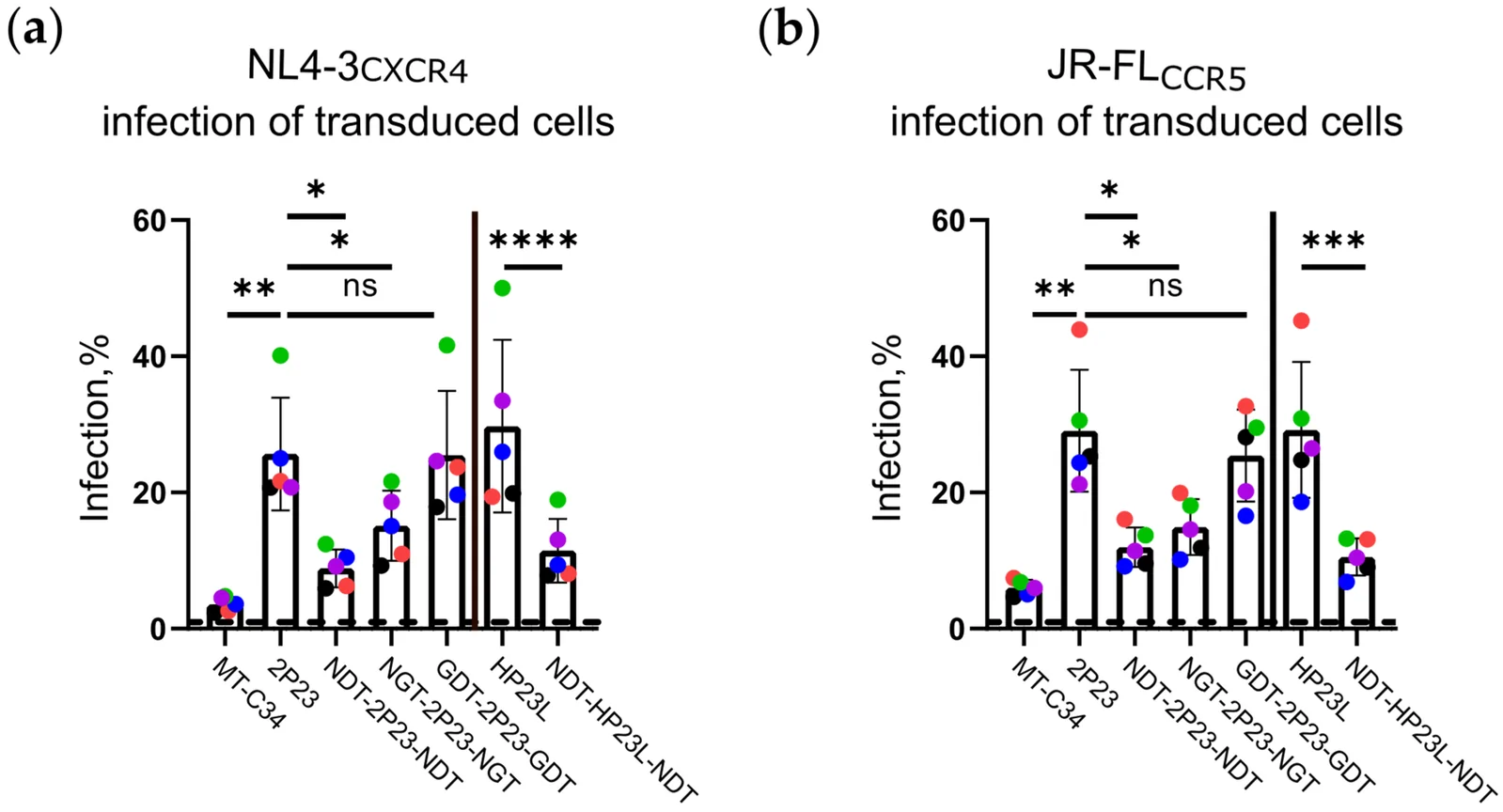
Protective properties of alternative peptide variants in cell co-culture infection assay. Cell co-culture assays evaluating cell-to-cell transmission of pseudoviruses harboring (**a**) the CXCR4-tropic NL4-3 envelope or (**b**) the CCR5-tropic JR-FL envelope in transduced CEM/CCR5 clone 8 cells expressing alternative surface peptides. Raji cells were electroporated with plasmids for pseudovirus assembly and co-cultured with target cells at a 1:1 ratio; 72 h after Raji electroporation, infection levels were assessed by measuring the luciferase reporter signal. Luciferase activity in peptide-expressing cells was normalized to that of control cells lacking the peptide. The black vertical line separates the HP23L data, which were analyzed separately from the 2P23 data. The dashed line indicates the background luciferase activity, determined by co-culturing pseudovirus-producing Raji cells with parental Raji cells. Data points from individual experiments are color-coded. For 2P23-variants statistical significance between multiple groups was evaluated using RM-ANOVA. To account for potential violations of the sphericity assumption, the Greenhouse–Geisser correction was applied, followed by Dunnett’s post hoc test for multiple comparisons against the 2P23 control. For HP23L-variants statistical significance was evaluated using a ratio paired *t*-test. *p*-values are categorized by significance thresholds as follows: (*) *p* < 0.05, (**) *p* < 0.01, (***) *p* < 0.001, (****) *p* < 0.0001, (ns)—not significant.

To evaluate the protective properties of the novel peptide variants, cell-to-cell virus transmission assays using the transduced cell lines were performed as described above (Figure 4a,b). The NGT-2P23-NGT variant mediated a statistically significant reduction in cell-to-cell transmission for both the NL4-3 (Figure 4a) and JR-FL (Figure 4b) pseudoviruses. Specifically, cells expressing the parental 2P23 control exhibited mean infection rates of 20% for NL4-3 and 25% for JR-FL, whereas the NGT-modified peptide lowered these rates to an average of 12% and 13%, respectively. Notably, these infection levels were higher than those observed for the NDT variant evaluated in parallel (7% for NL4-3 and 10% for JR-FL). These data indicate that both engineered variants (NDT and NGT) have increased protective properties as compared to 2P23. The infection rates in cells expressing the GDT-2P23-GDT control did not differ significantly from the 2P23, yielding values of 19% and 21%, respectively (Figure 4a,b) thus confirming the role of N-glycosylation in increased membrane expression and protective activity.

For both viral envelopes tested, the NDT-HP23L-NDT variant demonstrated significantly enhanced protective efficacy compared to the unmodified HP23L peptide (Figure 4a,b). The parental HP23L variant allowed infection levels of 23% with the NL4-3 envelope and 25% with the JR-FL envelope, whereas its glyco-engineered counterpart restricted viral entry to 9% for both envelopes. To confirm comparable pseudoviral infection efficiencies across generated cell lines, control assays using the VSV-G envelope were performed, demonstrating that all target cell lines were transduced with similar efficiencies regardless of the surface peptide variant (Supplementary Figure S4f).

## 3. Discussion

In this study, we engineered potential N-glycosylation sites into the sequence of the HIV-1 fusion inhibitor peptide 2P23 to enhance its cell-surface density and thereby boost its protective efficacy. N-linked glycosylation is one of the most prevalent post-translational protein modifications, wherein the oligosaccharyltransferase complex transfers a core oligosaccharide Glc2Man9GlcNAc2 onto an asparagine residue. This glycan chain transfer occurs co-translationally. Subsequently, carbohydrate chain maturation proceeds within the endoplasmic reticulum, mediated by glycosidases, mannosidases, and lectin chaperones [37]. This modification plays a critical role at various stages of protein biogenesis and function, including folding, quality control, and downstream stability [37]. Specifically, N-glycosylation serves a pivotal function in extending the half-life of membrane proteins by protecting them against proteolytic degradation [38,39] and denaturing agents [40]. We hypothesized that this modification could prolong the residence time of the 2P23 peptide on the plasma membrane thereby increasing its protective potency.

To modify the 2P23 peptide, two target locations at its N- and C-termini were selected. Expression of a panel of variants—specifically 2P23, NDT-2P23, 2P23-NDT, and NDT-2P23-NDT—in our cell lines demonstrated that the incorporation of NDT motifs, particularly when flanking both termini, consistently enhanced cell-surface expression levels (Figure 1c,d). Treatment with tunicamycin, an inhibitor of N-linked glycosylation, suggested that the observed accumulation effect for the 2P23-NDT and NDT-2P23-NDT constructs is indeed glycosylation-dependent (Figure 1e). Interestingly, while all three NDT-variants showed significantly increased plasma membrane levels compared to 2P23, only 2P23-NDT and NDT-2P23-NDT peptide levels were tunicamycin-dependent. We suggest that different N-glycosylation sites can exhibit variable site occupancy—defined as the percentage of molecules carrying the modification at a given position—depending on the adjacent amino acid residues [41]. Presumably, the N-terminal NDT variant is less prone to this modification than its C-terminal counterpart. In the double NDT variant, both modifications exert a cumulative effect, explaining the observed changes in peptide surface levels. However, we cannot rule out that the NDT motif within the NDT-2P23 variant influences peptide surface levels independently of N-glycosylation.

Notably, direct verification of glycan occupancy and its specific glycoform structure will require further experimental validation using PNGase F digestion [42] and mass spectrometry analysis [43]. To indirectly validate these findings, in the second part of the study, a non-glycosylatable GDT-2P23-GDT control variant was evaluated in parallel with NDT-2P23-NDT. In this control, the critical asparagine residue directly involved in carbohydrate conjugation was substituted with glycine. Compared to the 2P23 peptide, this mutant variant exhibited either lower (Figure 3b) or comparable (Figure 3c) cell-surface density and, reflecting the behavior of 2P23, remained unaffected by tunicamycin treatment (Figure 3f). Although tunicamycin induces endoplasmic reticulum stress and can trigger apoptotic or compensatory regulatory pathways in lymphoid cells [44,45], its non-specific cytotoxic effects are unlikely to explain our findings. Crucially, the differential responses observed between the NDT variants and GDT control demonstrate that this effect is biologically specific and glycosylation-dependent. Taken together, these data provide strong evidence supporting the presence of N-glycosylation within the NDT-2P23-NDT peptide.

Functional cell-coculture infection assays evaluating the inhibition of HIV-1 pseudovirus cell-to-cell transmission using the CXCR4-tropic NL4-3 and CCR5-tropic JR-FL strains demonstrated that the NDT-2P23-NDT peptide exhibited the most potent protective activity among the NDT-modified variants when delivered via stable lentiviral transduction (Figure 2a,b). This supports the hypothesis that the N- and C-terminal NDT modifications exert a cumulative effect, where a single modification is insufficient to enhance protective activity.

Although our current hypothesis is that elevated plasma membrane expression of the modified peptides is the primary driver of their enhanced protective properties, alternative contributing mechanisms cannot be ruled out. For instance, N-glycosylation can increase the local density of a modified peptide in close proximity to HIV coreceptors via galectin lattice formation [46]. Moreover, the NDT motif—either in its glycosylated or non-glycosylated state—could directly influence the peptide’s biochemical interaction with gp41. These hypotheses require extensive experimental validation, such as evaluating soluble forms of the modified peptides to independently determine their intrinsic inhibitory potency, completely decoupled from cell-surface expression levels.

In contrast to transduced cell lines, no statistically significant differences were observed between the various targeted knock-in cell lines (Figure 2c,d). This phenomenon can be explained by the fact that the peptide levels (reflected by MFI) were significantly higher in the knock-in cell lines than in their lentiviral counterparts. For instance, the surface density of the parental 2P23 peptide was approximately 8-fold higher in the knock-in cells. In the case of the CXCR4-tropic NL4-3 strain, additional protection is conferred by the concurrent *CXCR4* knockout, which is highly enriched within the sorted knock-in cell population. Ultimately, peptide membrane expression, in cooperation with the *CXCR4* knockout for the NL4-3 strain, likely saturated the protective threshold in our pseudoviral assay, masking any further incremental improvements among the NDT variants. Consequently, a critical next step in validating the therapeutic potential of the NDT-2P23-NDT constructs will be to confirm these findings using an infection model challenged with replication-competent HIV-1.

In addition to the NDT-2P23-NDT peptide variant, we evaluated an alternative construct, NGT-2P23-NGT, which possessed a higher predicted glycosylation score according to the NetNGlyc-1.0 web tool [27]. However, in both the transduced and targeted knock-in cell lines, this variant exhibited lower cell-surface expression levels than NDT-2P23-NDT (Figure 3b,c). Furthermore, the NGT-peptide variant demonstrated greater stability under tunicamycin treatment, showing a much smaller decrease in surface expression compared to NDT-2P23-NDT (Figure 3f). Taken together, these data suggest that the specific structural and local sequence context of the peptide may critically influence glycosylation efficiency that cannot be fully predicted by computational tools, underscoring the absolute necessity of experimental validation for in silico models.

We next sought to translate the successful sequence optimization strategy developed for 2P23 to a structurally similar fusion inhibitor, HP23L, which had also demonstrated poor inhibitory efficacy in the previous study [11]. Mirroring the trends observed for the 2P23/NDT-2P23-NDT pair, the engineered NDT-HP23L-NDT variant exhibited significantly higher cell-surface expression levels (Figure 3d,e) and mediated more potent inhibition of viral infection (Figure 4a,b). While this successful generalization indicates that N-glycosylation can be utilized to enhance peptide expression, this effect likely relies on the specific CD52-derived presentation context. Therefore, further studies are required to determine whether this approach can be successfully scaled and extended to alternative molecular scaffolds.

Previous studies demonstrated the efficacy of several membrane expression strategies for the C46 [47,48], C34 [21,22], and 2P23 [49] peptides. Elevating the cell-surface expression levels of the C46 peptide has been reported to confer an enhanced functional efficacy [47,48]. Our findings extend these data through a new strategy for increasing cell-surface peptide levels, which relies on embedding N-glycosylation motifs within the peptide sequence. Furthermore, we uncovered an expression-dependent trend for 2P23 glycosylation variants that, to our knowledge, has not been previously evaluated.

Interestingly, N-glycosylation can affect soluble and membrane-bound fusion inhibitors differently. While terminal glycosylation can enhance the pharmaceutical profiles of entry inhibitors like enfuvirtide [50,51], introducing a native N-glycan into soluble C34 at position 10 (corresponding to the residue N126 within the full-length gp41 sequence) slightly reduces anti-HIV activity, likely by destabilizing its interaction with the NHR [52]. This specific residue is tightly linked to viral fitness, as the secondary resistance substitution N126K is known to stabilize the endogenous viral six-helix bundle core [53]. We hypothesize that for membrane-bound peptides, the benefit of increased plasma membrane expression levels can substantially outweigh any reduced affinity for gp41 binding.

From a translational perspective, our strategy aligns with current methodologies for generating HIV-resistant cells, utilizing either lentiviral transduction or CRISPR/Cas9-mediated knock-in. As demonstrated previously, CD4^+^ T cells engineered via both approaches to express the membrane-anchored MT-C34 peptide confer a robust selective survival advantage over unmodified cells when challenged with replication-competent HIV-1 isolates [11]. Similarly, recent study demonstrated that delivering the 2P23 fusion inhibitor via lentiviral transduction efficiently protected both CD4^+^ T cells and CD34^+^ HSPCs from robust HIV-1 infection [54]. Next steps in our research will involve the production of third-generation lentiviral vectors encoding the newly optimized, glycosylated peptide variants, followed by functional evaluation in both CD4^+^ T and CD34^+^ HSPC cell populations using replication-competent HIV-1. Translating the knock-in protocol into primary human cells requires enhancing targeted editing levels, as current efficiencies remain a limiting barrier [55]. Furthermore, CRISPR/Cas9 integration into the *CXCR4* locus is suitable only for mature CD4^+^ T cells, as CXCR4 is required for the maintenance of HSPCs. To enable the genetic modification of HSPCs, this peptide knock-in strategy must be expanded to alternative loci, most notably the *CCR5* gene.

Because clinical data on gene therapies based on gp41-derived peptides are limited, the target cell percentage required for a functional cure can be inferred from CCR5-knockout clinical trials. These trials demonstrate that current levels of modified peripheral blood CD4^+^ T cells following engraftment are insufficient for viral control in the absence of cART [13,56,57]. Furthermore, murine models suggest that achieving viral control requires a frequency of greater than 90% of CCR5-negative cells within the engrafted HSPC pool [57]. Consistent with these findings, clinical evaluations of the gp41-derived peptides C34 and C46 revealed an insufficient number of HIV-resistant cells in patients’ peripheral blood [22,23]. Notably, following a clinical trial of C34 which indicated limited cellular persistence, the authors discovered that anchoring C34 to the N-terminus of CXCR4 likely induced aberrant CXCR4 signaling, resulting in T-cell dysfunction [23]. This underscores the critical importance of scaffold selection for therapeutic peptides. To address these limitations, maximize viral suppression, and mitigate the risk of viral escape, gp41-peptides are likely best suited for combination gene therapy approaches [58,59]. For instance, a single vector can be engineered to deliver three synergistic anti-HIV factors: the C46 peptide, a short hairpin RNA (shRNA) for *CCR5* knockdown, and a CD4-based chimeric antigen receptor designed to eliminate HIV-1-infected cells [59].

While combination designs incorporating gp41-derived peptides aim to replicate the functional cure achieved by the gold-standard *CCR5*-Δ32 HSPC transplantation model [19], their clinical translation faces unique ethical and regulatory hurdles [60]. Because modern cART—including long-acting regimens—has transformed HIV into a manageable chronic condition, the risk-benefit ratio of intensive gene therapies remains acceptable primarily for cART non-responders or patients with AIDS-related malignancies [60]. Consequently, a direct clinical comparison between emerging gene therapies and established cART standards is currently premature.

In the present study, we evaluated the protective properties of our modified peptides against two benchmark HIV strains: the CXCR4-tropic laboratory strain NL4-3 and the CCR5-tropic primary isolate JR-FL. However, given the extensive structural diversity of gp41 and the possibility that N-glycosylation or the NDT motifs themselves could modulate peptide binding to the NHR, these engineered membrane-anchored constructs must be evaluated against an expanded repertoire of genetically diverse strains and escape mutants conferring 2P23 resistance [8]. Additionally, long-term cultivation experiments are indispensable to fully determine the resistance kinetics and durable efficacy of these anchored peptides [61].

In conclusion, this study demonstrates that introducing flanking NDT motifs significantly enhances the cell-surface density and antiviral efficacy of the HIV-1 fusion inhibitor 2P23. Tunicamycin assays confirmed that this surface accumulation relies on active N-linked glycosylation. These findings were validated using an alternative glycosylatable NGT motif and a non-glycosylatable GDT control. Notably, experimental data overturned computational predictions for the alternative NGT motif, highlighting the necessity of experimental validation over predictive modeling. The optimized NDT-2P23-NDT variant mediated potent inhibition against both CXCR4- and CCR5-tropic HIV-1 strains during cell-to-cell transmission. This engineering strategy was successfully applied to a structurally related peptide, HP23L. Collectively, engineering N-glycosylation sites within a CD52 framework provides a versatile strategy for optimizing membrane-bound antiviral peptides, offering seamless compatibility with both transduction and CRISPR/Cas9-mediated knock-in in the development of HIV gene therapies.

## 4. Materials and Methods

### 4.1. Cell Culture

Human embryonic kidney 293T cells were obtained through the NIH AIDS Research and Reference Reagent Program. CEM/CCR5 clone 8 cells were generated by lentiviral transduction of the CCRF-CEM cell line (Cellosaurus CVCL_0207, purchased from the ATCC, Manassas, VA, USA) with human CCR5 cDNA [35]. All cell lines were cultured in DMEM/F12 (PanEco, Moscow, Russia) supplemented with 10% fetal bovine serum (#F800821, Globe Kang, Qinhuangdao, China), 4 mM L-glutamine (PanEco, Moscow, Russia), and 10 µg/mL gentamycin (PanEco, Moscow, Russia) at 37 °C in a 5% CO_2_ humidified atmosphere.

### 4.2. Plasmid Construction

All the plasmids used and generated in this study are shown in Table S1. Oligonucleotides used for cloning are listed in Table S2. All restriction endonucleases and other enzymes used for cloning are from SibEnzyme (SibEnzyme, Novosibirsk, Russia) if not stated otherwise. Nucleotide sequences of all plasmid constructs were verified by Sanger sequencing at Evrogen (Evrogen, Moscow, Russia). All oligonucleotides were synthesized by Evrogen. Plasmid DNA was isolated using the Plasmid Midiprep 2.0 kit (#BC124, Evrogen). DNA fragments from agarose gel were purified using the Cleanup S-Cap kit (#BC041L, Evrogen).

### 4.3. Construction of Peptide-Expressing Lentiviral Plasmids

The lentiviral plasmids pUCHR-mClover-2P23, pUCHR-mClover-MT-C34 and pUCHR-mClover-HP23L were previously described in the study by Maslennikova et al. [11] as pUCHR-mClover-smAID-P2A-2P23, pUCHR-mClover-smAID-P2A-MT-C34, and pUCHR-mClover-smAID-P2A-HP23L, respectively. Plasmids harboring the modified versions of 2P23 were generated via sequential PCR amplification of two fragments followed by their assembly using overlap extension PCR. All amplifications herein were performed using Pfu DNA polymerase (SibEnzyme, E363, 5000 U/mL). The first PCR fragment was amplified from the template pUCHR-mClover-smAID-P2A-CD5HA2 [11] with 5′-mClover-AID#2 primer and a 3′-oligonucleotide containing a terminal modification encoding the corresponding peptide fragment (primers 3′2P23, 3′NDT-2P23, 3′NGT-2P23, 3′GDT-2P23, and 3′NDT-HP23L, Table S2). The second fragment was amplified using a 5′-oligonucleotide with a terminal modification (primers 5′2P23, 5′2P23-NDT, 5′2P23-NGT, 5′2P23-GDT, and 5′HP23L-NDT, Table S2) and the 3′-PspXI-LTR primer. The resulting PCR products were diluted 50-fold, and 1 µL of the dilution was subsequently used for the overlap extension PCR with the 5′-mClover-AID#2 and 3′-PspXI-LTR primers. The final PCR products were digested with AfeI and PspXI restriction endonucleases and cloned into the corresponding sites of the pUCHR-mClover-smAID-P2A-CD5HA2 vector, treated with shrimp alkaline phosphatase (SibEnzyme, E365, 5000 U/mL).

### 4.4. Construction of Donor Plasmids for Peptide Knock-In into the CXCR4 Exon 2 Locus

To construct the universal donor vector pBluescript-X4ex2donor(Cas9)-short_template, which is designed for the knock-in of peptide sequences into exon 2 of the CXCR4 gene, the CXCR4 homology arms were amplified on the template of pJET-donor_MT-C34 plasmid [11]. Specifically, the 634-bp 5′-homology arm was amplified with the 5′-X4-arm499bp-fwd and 3′BbsI-P2A-rev primers, and the 577-bp 3′-homology arm was amplified with the 5′BbsI-3′arm472bp-fwd and 3′-X4-arm472bp-rev primers. The resulting PCR products were used for the overlap extension PCR as described above with the 5′-X4-arm499bp-fwd and 3′-X4-arm472bp-rev primers, yielding an 1181-bp product. Next, the PCR product was purified via gel electrophoresis, treated with T4 polynucleotide kinase, and cloned into the EcoRV site of pBluescript KS(+) vector (Stratagene, Santa Clara, CA, USA). The resulting donor vector contained the CXCR4 homology arms, a P2A sequence, a region flanking BbsI restriction sites for the insertion of peptide sequences, a partial CD52 sequence, and a polyA signal.

To generate the donor plasmids for knock-in into *CXCR4* locus, the peptide sequences (2P23, HP23L, NDT-2P23, 2P23-NDT, NDT-2P23-NDT, NGT-2P23-NGT, GDT-2P23-GDT, NDT-HP23L-NDT, MT-C34) were amplified by PCR with 5′BbsI-CD52-X2P23 and 3′BbsI-X2P23 primers from the respective pUCHR-mClover-peptide lentiviral plasmids described above. PCR products were digested with BbsI restriction endonuclease and cloned into the BbsI site pBluescript-X4ex2donor(Cas9)-short_template vector.

Additional cloning details are available upon reasonable request.

### 4.5. Lentivirus Production

To produce lentiviruses, 293T cells were seeded at a density of 10^6^ cells per 60-mm dish in 5 mL of full medium. After 24 h, the cells were transfected with the pUCHR-mClover plasmid encoding the corresponding peptide, pCMV-deltaR8.2 (Addgene, Watertown, MA, USA; plasmid #12263), and pCMV-VSV-G (Addgene; plasmid #8454, [62]) at a ratio of 4:3:1, respectively. Transfection was performed using 2.5 µg of total DNA and 5 µL of GenJect-39 transfection reagent (Molecta, Moscow, Russia) in Opti-MEM medium (#31985070, ThermoFisher Scientific, Waltham, MA, USA) according to the manufacturer’s protocol. The culture medium was replaced 4 h post-transfection. After 48 h, the lentivirus-containing supernatant was harvested, centrifuged at 350× *g* for 5 min, and filtered through a 0.45-µm PVDF filter (#FPV403025, Guangzhou Jet Bio-Filtration Co., Ltd., Guangzhou, China). The filtrate was then centrifuged at 21,000× *g* for 2.5 h at 4 °C. The lentiviral particles were concentrated 10-fold and stored at −70 °C.

### 4.6. Lentiviral Transduction

For transduction, 10^5^ CEM/CCR5 clone 8 cells were seeded per well of a 24-well plate in 500 µL of culture medium and transduced the following day with a 10-fold concentrated lentivirus at a dose yielding less than 30% of mClover-positive cells. On the following day, half of the culture medium was replaced with fresh medium. At 48 h post-transduction, the mClover expression levels were evaluated using flow cytometry.

### 4.7. Electroporation

For knock-in of peptide sequences into exon 2 of the *CXCR4* gene, 1.2 × 10^6^ CEM/CCR5 clone 8 cells were electroporated with 3 µg of the pcDNA3.3-hSpCas9 plasmid (#41815, Addgene, [63]), 1 µg of the pKS-U6-sgRNA-X4ex2 plasmid [11] encoding a guide RNA (gRNA) targeting exon 2 of the *CXCR4* gene (cacttcagataactacaccgAGG—spacer and PAM), and 5 µg of the pBluescript-X4ex2donor(Cas9)-peptide donor vector (described above). Electroporation was performed using the Neon Transfection System (Invitrogen, Thermo Fisher Scientific, Waltham, MA, USA) with 100-µL tips under the following conditions: 1230 V, 40 ms, and 1 pulse. Surface levels of the peptides were evaluated by flow cytometry on the day 5 post-electroporation.

### 4.8. Flow Cytometry and Cell Sorting

To evaluate the surface expression of 2P23 and its modified variants, cells were stained with rabbit polyclonal anti-2P23 antibodies (0.52 mg/mL) described previously [11]. The primary antibodies were diluted 1:1000 in PBS in the presence of bovine serum albumin (BSA) of 0.01 mg/mL. Cells were washed once with PBS and incubated with the primary antibodies for 30 min at 4 °C. Following incubation, the cells were washed twice with PBS and subsequently stained with secondary antibodies. For the detection of 2P23 and its modifications, Alexa Fluor 647-conjugated goat anti-rabbit IgG secondary antibodies (Thermo Fisher Scientific, A-21244, 1:300) were used. Cells were then incubated at 4 °C for 30 min, washed twice with PBS, and fluorescence was analyzed using a CytoFLEX S flow cytometer (Beckman Coulter, Inc., Brea, CA, USA).

All other immunostaining experiments were performed similarly, varying only in the primary and secondary antibody combinations.

Cells expressing MT-C34, HP23L, and NDT-HP23L-NDT peptides were stained with the respective undiluted mouse hybridoma supernatants, as described previously [11], followed by Alexa Fluor 647-conjugated goat anti-mouse IgG secondary antibodies (ThermoFisher Scientific, A-21235, 1:300). In tunicamycin experiments, rabbit polyclonal antibodies (1:1000) were used instead: cells expressing MT-C34 were stained in the presence of 0.1 mg/mL BSA, whereas cells expressing HP23L were stained with 0.5 mg/mL BSA [11].

To assess the surface expression of CD4, CCR5, and CXCR4, cells were incubated with primary mouse monoclonal antibodies against CD4 (1:200; kindly provided by Dr. A.V. Filatov, Institute of Immunology, FMBA, Moscow, Russia), CXCR4 (clone 12G5, sc-12764, Santa Cruz Biotechnology, Inc., Dallas, TX, USA, 1:100), or CCR5 (555991, BD Biosciences Pharmingen, San Jose, CA, USA, 1:250) followed by aforementioned Alexa Fluor 647-conjugated goat anti-mouse IgG secondary antibodies. To evaluate CXCR4 surface levels on Raji cells, the cells were stained with a PE-conjugated mouse anti-human CXCR4 monoclonal antibody (1:100, #E-AB-F1157D, Elabscience, Houston, TX, USA).

Transduced CEM/CCR5 clone 8 cells expressing individual peptides were enriched by three rounds of FACS based on the mClover fluorescent signal. Similarly, CEM/CCR5 clone 8 cells harboring the peptide knock-in into exon 2 of the *CXCR4* gene were stained for the respective peptides and sorted by FACS on the basis of the peptide-specific fluorescent signal. All resulting cell lines achieved a purity of ≥95% mClover-positive cells for the transduced lines and ≥95% peptide-positive cells for the knock-in lines (Supplementary Figures S1a,e and S4a). Cell sorting was performed on a Sony MA900 cell sorter using the FITC and APC-A channels, respectively. Cell sorting was conducted using the equipment of the Institute of Gene Biology, Russian Academy of Sciences (IGB RAS), supported by the Ministry of Science and Higher Education of the Russian Federation.

### 4.9. Experiments with the Glycosylation Inhibitor Tunicamycin

To evaluate the effects of N-glycosylation, 0.5 × 10^6^ CEM/CCR5 clone 8 cells were seeded in 1.5 mL of culture medium per well of a 12-well plate and cultured in the medium containing 0.5 µg/mL tunicamycin (T-7765, Sigma Chemical Co., St. Louis, MO, USA), or 0.5 µg/mL dimethyl sulfoxide (DMSO) (PanEco, F135) as the vehicle control, or without any additives. After 5 days of culture, cell membrane expression levels of the peptides were quantified by flow cytometry. For data analysis, peptide median fluorescence intensity (MFI) values from DMSO-treated or tunicamycin-treated samples were normalized against those from corresponding control cells cultured in unmodified complete medium using the following formulas: MFI_Tun_/MFI_unmodified control_ and MFI_DMSO_/MFI_unmodified control_.

### 4.10. Cell Coculture Infection

To perform the cell-to-cell viral infection assays utilizing the intron-regulated reporter vector inLuc [36], a previously described protocol was adopted [11]. Briefly, 2 × 10^6^ Raji cells were electroporated using the Neon system (1350 V, 30 ms, 1 pulse) with a mixture of 2 µg of pCMV-ΔR8.2, 3 µg of pUCHR-inLuc-mR, and 1 µg of an HIV envelope-encoding vector (either pIIINL4env encoding the HIV-1 NL4-3 envelope [64] or pJRFL encoding the HIV-1 JR-FL envelope [65]) or pCMV-VSV-G. Following electroporation, the cells were cultured in antibiotic-free medium for 6 h, then these producer cells were mixed with target cells at a 1:1 ratio. For the co-culture, 0.5 × 10^6^ Raji cells were combined with 0.5 × 10^6^ control CEM/CCR5 clone 8 cells or peptide-expressing cells, seeded into 12-well plates in 2 mL of medium, and incubated for 48 h. Sixteen hours prior to detection, phorbol 12-myristate 13-acetate was added to the cultures at a final concentration of 20 nM to enhance reporter gene expression in the infected cells. The total duration from Raji electroporation to the detection of luciferase activity spanned 72 h. To detect the bioluminescent signal, the cells were harvested by centrifugation, resuspended in 110 µL of Glo Lysis Buffer from the Bright-Glo Luciferase Assay System (Promega, #E2620, Madison, WI, USA), and incubated for 10 min at room temperature. The lysates were then centrifuged at 15,000× *g* for 5 min. A 100-µL aliquot of the cleared lysate was mixed with 100 µL of luciferin substrate (Promega, #E2620, USA), vortexed, and the luminescence signal was measured using a GloMax-Multi Jr single-tube multimode reader (Promega, Madison, WI, USA).

### 4.11. Statistical Analysis

Data presented as mean ± standard deviation (SD) from at least three independent biological replicates were analyzed in GraphPad Prism 8.0.1 Software (Boston, MA, USA). In all graphical representations, individual data points from independent experiments are color-coded to show biological consistency across conditions.

Statistical significance between multiple groups was evaluated using repeated-measures analysis of variance (RM-ANOVA). To account for potential violations of the sphericity assumption, the Greenhouse–Geisser correction was applied, followed by Dunnett’s post hoc test for multiple comparisons against the 2P23 control. Pairwise statistical comparisons between two related groups (e.g., tunicamycin-treated versus DMSO-treated samples, or specific HP23L-derived variant evaluation) were performed using a ratio paired *t*-test. *p*-values are categorized by significance thresholds as follows: (*) *p* < 0.05, (**) *p* < 0.01, (***) *p* < 0.001, (****) *p* < 0.0001, (ns)—not significant.

To calculate the correlation between the mean infection rate and the mean peptide MFI on the cell line, Pearson’s correlation coefficient (*r*) was used (Figure S3c).

## Data Availability

Data are contained within the article and Supplementary Materials.

## Supplementary Materials

The following supporting information can be downloaded at: https://www.mdpi.com/article/10.3390/ijms27219695/s1.

## Abbreviations

The following abbreviations are used in this manuscript:

HIV	Human Immunodeficiency Virus
CCR5	C-C chemokine receptor type 5
CXCR4	C-X-C chemokine receptor type 4
BSA	Bovine Serum Albumin
FACS	Fluorescence-Activated Cell Sorting
MFI	Median Fluorescence Intensity
