# Supplementary material for: Modification of the HIV-1 Fusion Inhibitor 2P23 via N-Glycosylation Sites Enhances Its Cell Membrane Level and Antiviral Activity
Source: Int J Mol Sci. 2026 Oct 29;27(21):9695. doi: 10.3390/ijms27219695 (PMC13763309; doi:10.3390/ijms27219695)
Supplement: Supplementary file 1 [file ijms-27-09695-s001.zip › ijms-4606975-supplementary.pdf]

Supplementary Information

Modification of the HIV-1 fusion inhibitor 2P23 via N-glycosylation sites enhances its cell membrane level and antiviral activity

Sofiia E. Borovikova, Anna V. Tvorogova, Mikhail V. Shepelev, and Natalia A. Kruglova

Supplementary figures

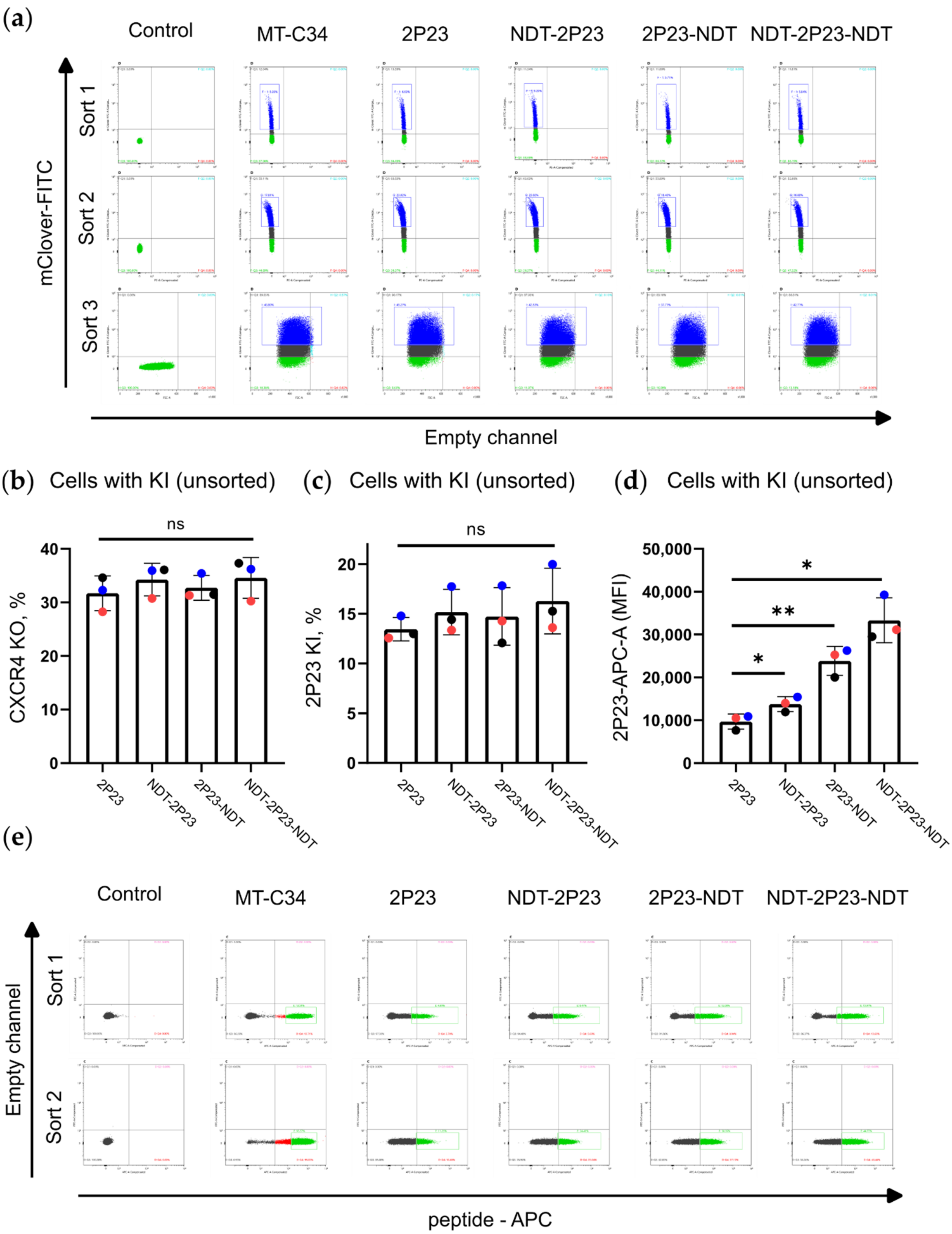

Figure S1. Cell-surface expression of NDT peptides. (a) Fluorescence-activated cell sorting (FACS) of CEM/CCR5 clone 8 populations transduced with NDT peptides. Cells were sorted based on mClover signal in the FITC channel. The sorting gate is highlighted in blue. b–d) Flow cytometry analysis of CEM/CCR5 clone 8 cells at day 5 post-electroporation with NDT peptide donors. Panels show (b) the percentage of CXCR4-knockout cells, (c) the percentage of knock-in cells, and (d) the median fluorescence intensity (MFI) of the peptide within the knock-in gate of unsorted cells. For panels from (b) to (d) statistical significance between multiple groups was

evaluated using RM-ANOVA. To account for potential violations of the sphericity assumption, the Greenhouse–Geisser correction was applied, followed by Dunnett's post hoc test for multiple comparisons against the 2P23 control. p-values are categorized by significance thresholds as follows: (*) $p < 0.05$, (**) $p < 0.01$, (***) $p < 0.001$, (****) $p < 0.0001$, (ns) - not significant. (e) FACS of CEM/CCR5 clone 8 cells with NDT peptide knock-in based on peptide expression in the APC-A channel. The sorting gate is highlighted in green.

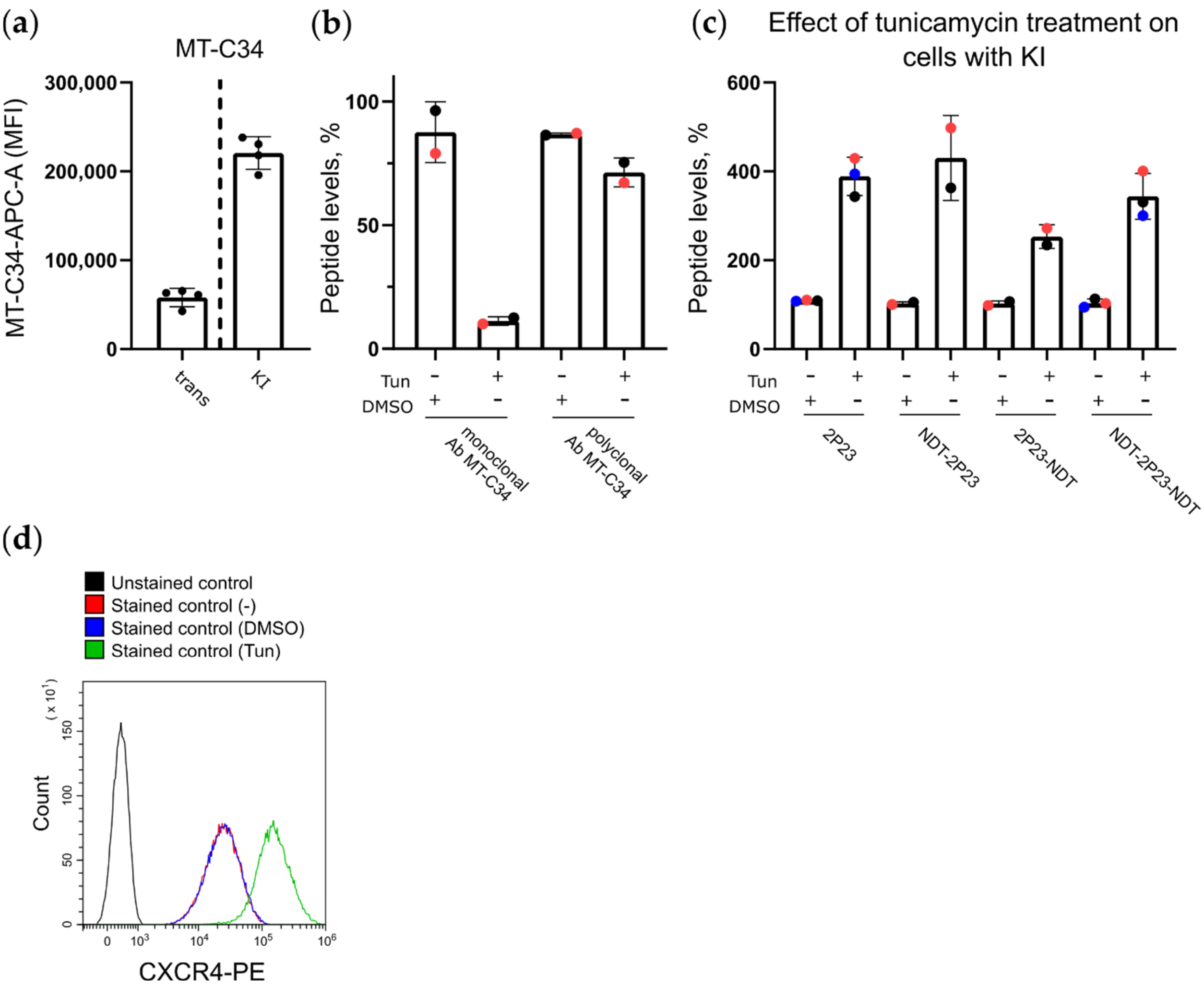

Figure S2. Experiments with tunicamycin. (a) Flow cytometry analysis of sorted transduced (trans) and peptide knock-in (KI) CEM/CCR5 clone 8 cells expressing the MT-C34 peptide. (b) Comparison of MT-C34 staining using monoclonal versus polyclonal antibodies following tunicamycin treatment in transduced MT-C34 CEM/CCR5 clone 8 cells. (c) Flow cytometry analysis of peptide levels under tunicamycin treatment in sorted KI CEM/CCR5 clone 8 cells. For (b, c), cells were cultured for 5 days in medium supplemented with either tunicamycin or DMSO, followed by surface peptide staining. The median peptide fluorescence intensity (MFI) in the APC channel was normalized to that of stained control cells cultured in unmodified medium. Tun, tunicamycin. (d) Flow cytometry analysis of CXCR4 HIV coreceptor expression in CEM/CCR5 clone 8 cells following tunicamycin incubation. Cells were cultured for 5 days with tunicamycin (Tun), DMSO vehicle control or left untreated in unmodified medium (-). The resulting histograms are overlaid on a single plot.

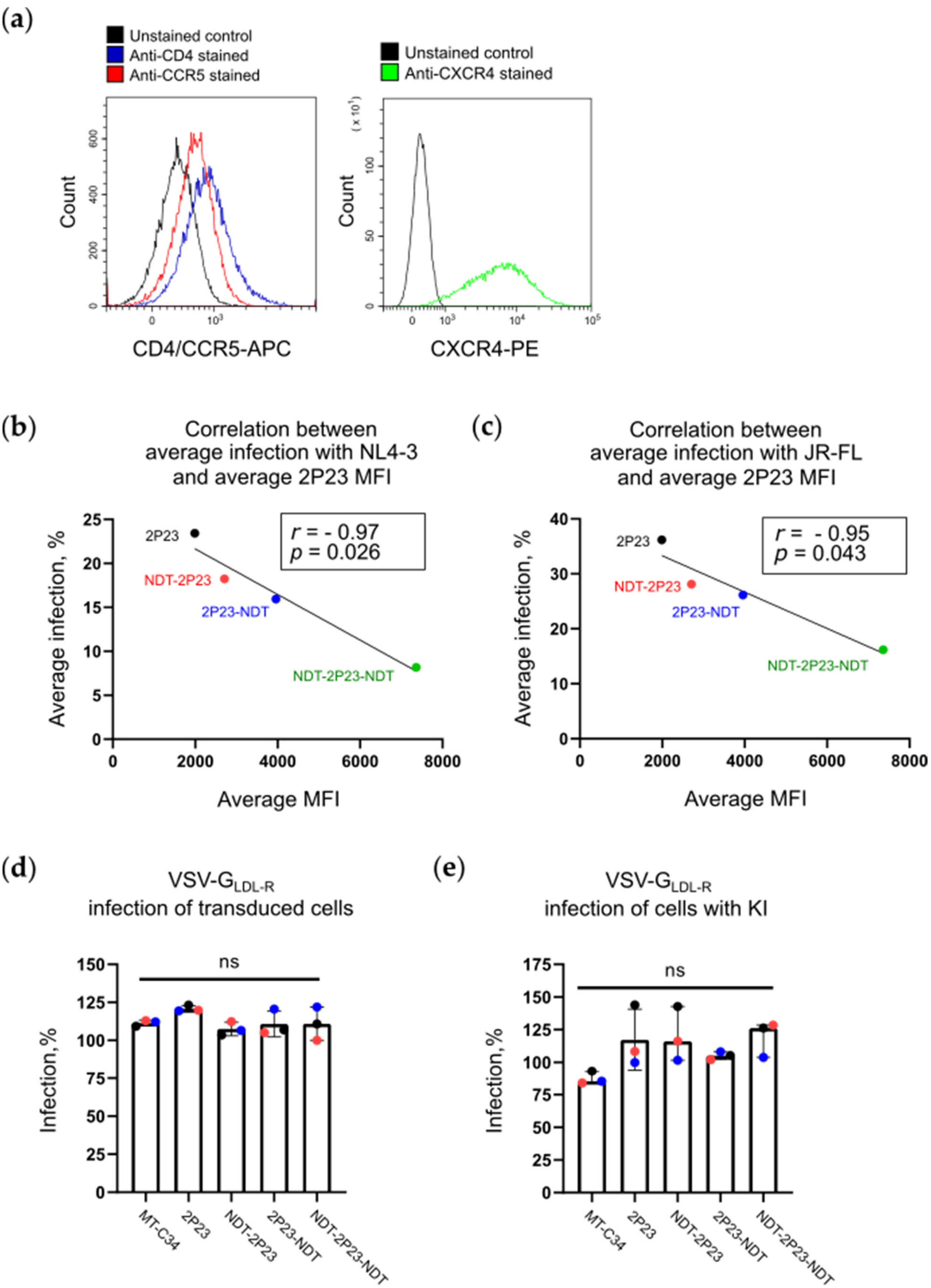

Figure S3. Cell-to-cell infection assays with NDT-variants. (a) Flow cytometry analysis of HIV receptors and coreceptors in Raji cells. b, c) Correlation between cell-to-cell infection rates and peptide MFI levels. Pearson correlation analysis between the average cell-to-cell infection rates (derived from the data in Figures 2a and 2b) and the average MFI of the peptides (derived from

the data in Figure 1c) for four 2P23-based peptides (2P23, NDT-2P23, 2P23-NDT, and NDT-2P23-NDT). The solid line represents the linear trendline (line of best fit). The Pearson correlation coefficient (*r*) and the corresponding *p*-value are indicated within the inset box. d, e) Cell co-culture assays evaluating cell-to-cell transmission of VSV-G-pseudotyped viruses in d) sorted transduced and e) sorted knock-in (KI) CEM/CCR5 clone 8 cells. For (d) and (e) panels statistical significance between multiple groups was evaluated using RM-ANOVA. *p*-values are categorized by significance thresholds as follows: (*) $p < 0.05$, (**) $p < 0.01$, (***) $p < 0.001$, (****) $p < 0.0001$, (ns) - not significant.

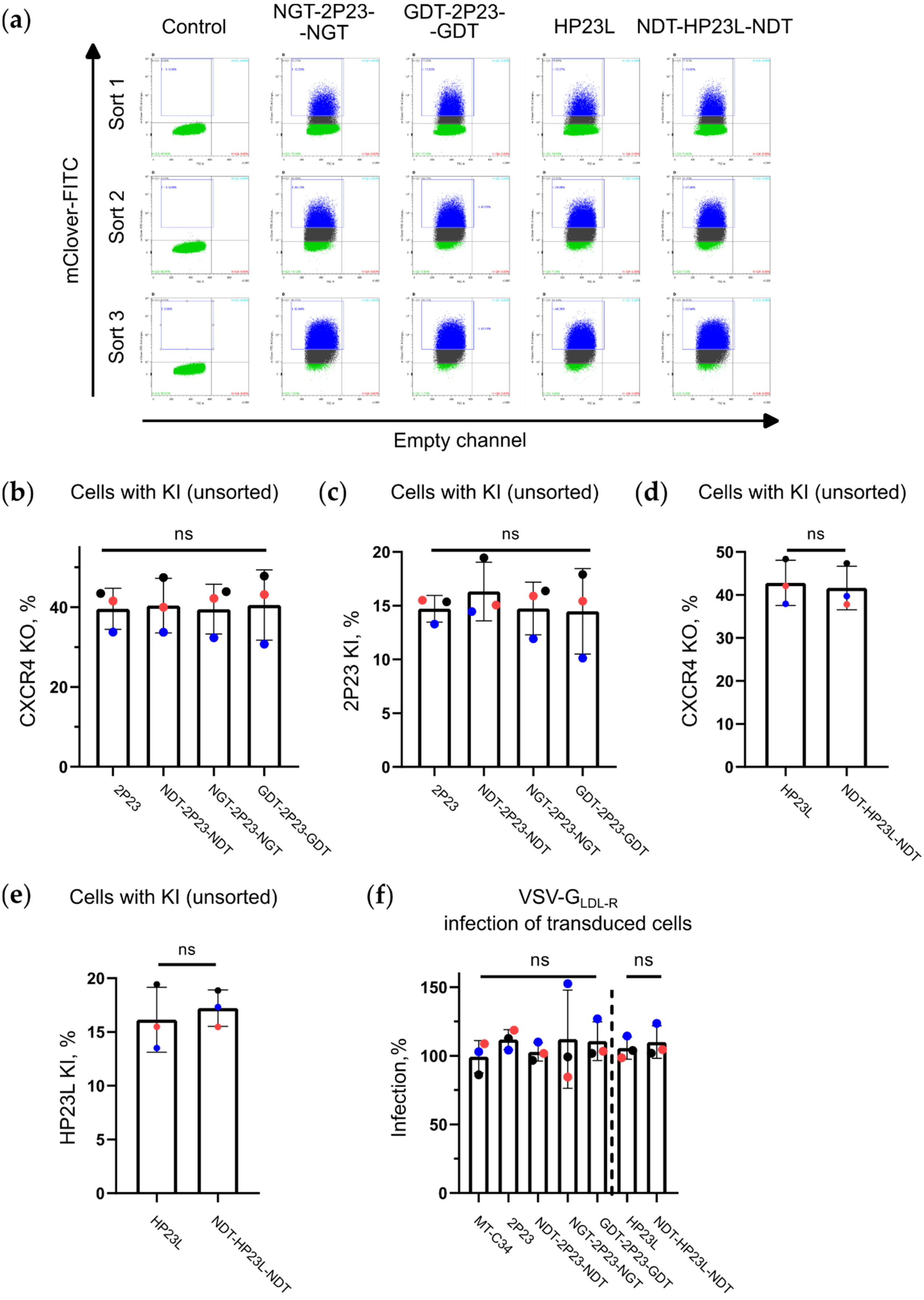

Figure S4. Cell surface expression and activity in cell-coculture infection of alternative peptide variants. (a) FACS of CEM/CCR5 clone 8 populations transduced with alternative peptide variants. Cells were sorted based on mClover signal in the FITC channel. The sorting gate is highlighted in blue. b–e) Flow cytometry analysis of CEM/CCR5 clone 8 cells at day 5 post-

electroporation with alternative peptide donors. Panels show b, d) the percentage of CXCR4-knockout cells and c, e) the percentage of peptide knock-in cells for b, c) 2P23-derived variants and d, e) HP23L-derived variants. (f) Co-culture assays evaluating the cell-to-cell transmission of VSV-G-pseudotyped viruses in sorted transduced CEM/CCR5 clone 8 cells expressing alternative cell-surface peptides. The dashed line indicates background luciferase activity from co-cultures of pseudovirus-producing Raji cells with parental Raji cells. For panels (b), (c) and partly (f) (2P23-variants only) statistical significance between multiple groups was evaluated using RM-ANOVA. For panels (d), (e) and partly (f) (HP23L-variants only) statistical significance was evaluated a ratio paired *t*-test. *p*-values are categorized by significance thresholds as follows: (*) $p < 0.05$, (**) $p < 0.01$, (***) $p < 0.001$, (****) $p < 0.0001$, (ns) - not significant.

Supplementary tables

Table S1. Plasmid constructs used in this study.

Plasmid	Source	Description
pUCHR-mClover-smAID-P2A-CD5HA2	[11]	Template for CD52 scaffold amplification
pUCHR-mClover-MT-C34	[11]	Lentiviral vector for MT-C34 peptide expression
pUCHR-mClover-2P23	[11]	Lentiviral vector for 2P23 peptide expression
pUCHR-mClover-NDT-2P23	This study	Lentiviral vector for NDT-2P23 peptide expression
pUCHR-mClover-2P23-NDT	This study	Lentiviral vector for 2P23-NDT peptide expression
pUCHR-mClover-NDT-2P23-NDT	This study	Lentiviral vector for NDT-2P23-NDT peptide expression
pUCHR-mClover-NGT-2P23-NGT	This study	Lentiviral vector for NGT-2P23-NGT peptide expression
pUCHR-mClover-GDT-2P23-GDT	This study	Lentiviral vector for GDT-2P23-GDT peptide expression
pUCHR-mClover-HP23L	This study	Lentiviral vector for HP23L peptide expression
pUCHR-mClover-NDT-HP23L-NDT	This study	Lentiviral vector for NDT-HP23L-NDT peptide expression
pCMV-VSVG	#8454, Addgene [62]	Plasmid expressing VSVG for lentivirus production
pCMV delta R8.2	#12263, Addgene	Packaging plasmid for lentivirus production
pcDNA3.3-hSpCas9	#41815, Addgene [63]	Plasmid expressing SpCas9
pKS-U6-sgRNA-X4ex2	[11], [35]	Plasmid expressing gRNA SpCas9 against *CXCR4* gene exon 2
pBluescript-X4ex2donor(Cas9)-short_template	This study	Template for creating donor plasmids with protective peptides for knock-in into *CXCR4* gene exon 2
pBluescript-X4ex2donor(Cas9)-MT-C34	This study	Donor plasmid for MT-C34 peptide knock-in into exon 2 of the *CXCR4* gene
pBluescript-X4ex2donor(Cas9)-2P23	This study	Donor plasmid for 2P23 peptide knock-in into exon 2 of the *CXCR4* gene
pBluescript-X4ex2donor(Cas9)-NDT-2P23	This study	Donor plasmid for NDT-2P23 peptide knock-in into exon 2 of the *CXCR4* gene

pBluescript-X4ex2donor(Cas9)-2P23-NDT	This study	Donor plasmid for 2P23-NDT peptide knock-in into exon 2 of the *CXCR4* gene
pBluescript-X4ex2donor(Cas9)-NDT-2P23-NDT	This study	Donor plasmid for NDT-2P23-NDT peptide knock-in into exon 2 of the *CXCR4* gene
pBluescript-X4ex2donor(Cas9)-NGT-2P23-NGT	This study	Donor plasmid for NGT-2P23-NGT peptide knock-in into exon 2 of the *CXCR4* gene
pBluescript-X4ex2donor(Cas9)-GDT-2P23-GDT	This study	Donor plasmid for GDT-2P23-GDT peptide knock-in into exon 2 of the *CXCR4* gene
pBluescript-X4ex2donor(Cas9)-HP23L	This study	Donor plasmid for HP23L peptide knock-in into exon 2 of the *CXCR4* gene
pBluescript-X4ex2donor(Cas9)-NDT-HP23L-NDT	This study	Donor plasmid for NDT-HP23L-NDT peptide knock-in into exon 2 of the *CXCR4* gene
pUCHR-inLuc-mR	[36], [11]	Plasmid encoding an intron-split inverted luciferase for cell-to-cell infection assay
pIIINL4env	[64]<br>kindly provided by Dr. Eric Freed, NCI-Frederick, USA	Plasmid expressing CXCR4-tropic HIV envelope NL4-3
pJRFLenv	[65]<br>generously provided by Dr. E. Hunter and Dr. J. Blackwell, Emory University, GA, USA	Plasmid expressing CCR5-tropic HIV envelope JR-FL

Table S2. Oligonucleotide primers.

Oligonucleotide	Sequence, 5′ → 3′	Description
5′2P23	GAAAAGAAGGTCGAGGAACTGGAGAAGAAAATCGAAGAGCTGCTCAAGTCAGCATCCAGCAACATAAG	Cloning of the NDT-2P23 peptide in the lentiviral plasmid
3′NDT-2P23	CAGTTCCTCGACCTTCTTTTCCCACTCTTCCCAGGTCATCTCGGTGTCGTTTGAGAGTCCAGTTTGTATCTG	Cloning of the NDT-2P23 and the NDT-2P23-NDT peptides in the lentiviral plasmid
5′2P23-NDT	GAAAAGAAGGTCGAGGAACTGGAGAAGAAAATCGAAGAGCTGCTCAAGAACGACACCTCAGCATCCAGCAACATAAG	Cloning of the 2P23-NDT and the NDT-2P23-NDT peptides in the lentiviral plasmid

3′2P23	CAGTTCCTCGACCTTCTTTTCCCACTCTTCCCAGGTCATCTCTGAGAGTCCAGTTTGTATCTG	Cloning of the 2P23-NDT peptide in the lentiviral plasmid
3′NGT-2P23	CAGTTCCTCGACCTTCTTTTCCCACTCTTCCCAGGTCATCTCGGTGCCGTTTGAGAGTCCAGTTTGTATCTG	Cloning of the NGT-2P23-NGT peptide in the lentiviral plasmid
5′2P23-NGT	GAAAAGAAGGTCGAGGAACTGGAGAAGAAAATCGAAGAGCTGCTCAAGAACGGCACCTCAGCATCCAGCAACATAAG	Cloning of the NGT-2P23-NGT peptide in the lentiviral plasmid
3′GDT-2P23	CAGTTCCTCGACCTTCTTTTCCCACTCTTCCCAGGTCATCTCGGTGTCGCCTGAGAGTCCAGTTTGTATCTG	Cloning of the GDT-2P23-GDT peptide in the lentiviral plasmid
5′2P23-GDT	GAAAAGAAGGTCGAGGAACTGGAGAAGAAAATCGAAGAGCTGCTCAAGGGCGACACCTCAGCATCCAGCAACATAAG	Cloning of the GDT-2P23-GDT peptide in the lentiviral plasmid
3′NDT-HP23L	GTATTCCTCGATCTTCTTTTCCCACTCTTCCCAGGTCAGCTCGGTGTCGTTTGAGAGTCCAGTTTGTATCTG	Cloning of the NDT-HP23L-NDT peptide in the lentiviral plasmid
5′HP23L-NDT	GGAAAAGAAGATCGAGGAATACACCAAGAAAATCGAAGAGATCCTCAAGAACGACACCTCAGCATCCAGCAACATAAG	Cloning of the NDT-HP23L-NDT peptide in the lentiviral plasmid
5′-mClover-AID#2	CTGTACAAGCCAGCCAAAC	Cloning of fusion peptides in lentiviral plasmid
3′-PspXI-LTR	CATTGGTCTTAAAGGTACTCGAG	Cloning of fusion peptides in lentiviral plasmid
5′-X4-arm499bp-fwd	TTTGCGCTTTAGGAGAATGAGT	Cloning of homology arms for donor plasmid template
3′BbsI-P2A-rev	TTGGCCGTCTTCTCGAAGACTTCGGTCCAGGATTCTCTTC	Amplification of homology arms for donor plasmid template cloning
5′BbsI-3′arm472bp-fwd	ACCGAAGTCTTCGAGAAGACGGCCAATGCCATAATCCAC	Amplification of homology arms for donor template plasmid pBluescript-X4ex2donor(Cas9)-short_template
3′X4-arm472bp-rev	ATCGGGAATAGTCAGCAGG	Amplification of homology arms for donor template plasmid pBluescript-X4ex2donor(Cas9)-short_template
5′BbsI-CD52-X2P23	GGTAGAAGACGGACCGAAGCGCTTCCTC	Cloning of fusion peptides into donor template plasmid pBluescript-X4ex2donor(Cas9)-short_template

3’BbsI-X2P23	AAATGAAGACCATTGGCCACGAA GAAAAGGA	Cloning of fusion peptides into donor template plasmid pBluescript-X4ex2donor(Cas9)-short_template
